# RNA m^6^A modification orchestrates the rhythm of immune cell development from hematopoietic stem cells to T and B cells

**DOI:** 10.3389/fimmu.2022.839291

**Published:** 2022-07-22

**Authors:** Chuanxiang Zhao, Guoying Xu, Xiaoxian Zhang, Yunfeng Ye, Weili Cai, Qixiang Shao

**Affiliations:** ^1^ Institute of Medical Genetics and Reproductive Immunity, School of Medical Science and Laboratory Medicine, Jiangsu College of Nursing, Huai’an, China; ^2^ Reproductive Sciences Institute, Jiangsu Key Laboratory of Medical Science and Laboratory Medicine, Department of Immunology, School of Medicine, Jiangsu University, Zhenjiang, China

**Keywords:** N^6^-methyladenosine, hematopoietic stem cell, T cell, B cell, RNA

## Abstract

RNA, one of the major building blocks of the cell, participates in many essential life processes. RNA stability is well-established to be closely related to various RNA modifications. To date, hundreds of different RNA modifications have been identified. N6-methyladenosine (m^6^A) is one of the most important RNA modifications in mammalian cells. An increasing body of evidence from recently published studies suggests that m^6^A modification is a novel immune system regulator of the generation and differentiation of hematopoietic stem cells (HSCs) and immune cells. In this review, we introduce the process and relevant regulatory mechanisms of m^6^A modification; summarize recent findings of m^6^A in controlling HSC generation and self-renewal, and the development and differentiation of T and B lymphocytes from HSCs; and discuss the potential mechanisms involved.

## 1 Introduction

RNA is widely acknowledged to play an extremely important role in living organisms. RNA stability largely depends on its modification, one of the most important post-transcriptional regulations (1). To date, more than 100 RNA modifications have been identified in eukaryotes (2). Among them, RNA methylation is one of the predominant forms, accounting for more than 60% of RNA modifications (2). N6-methyladenosine (m^6^A), a modification occurring at the 6th position of adenine (A) bases, is the most prevalent internal RNA modification (3). The first report about m^6^A was published in 1974 (4). However, due to the lack of m^6^A-mapping methods, research on m^6^A has been in the initial stage for a long time. Until 2012, m^6^A-specific methylated RNA immunoprecipitation with next-generation sequencing (MeRIP-seq) was established and an extensive study of m^6^A modification was conducted by transcriptome analysis (5, 6). Since then, a mounting number of studies have substantiated m^6^A modification in various cell types, unveiling the mystery of m^6^A in a wide range of physiological and pathological processes, such as stem cell differentiation (7), the maintenance of pluripotency in embryonic development (8), X chromosome inactivation (9), virus replication (10), and the generation, development, invasion, metastasis, and drug resistance of cancer cells (11).

Overwhelming evidence substantiates that RNA m^6^A modification represents a significant role in the immune system. Here, we introduce the process and molecular mechanism involved in RNA m^6^A modification and summarize recent investigations highlighting the crucial regulatory role of RNA m^6^A modification in hematopoietic stem cell (HSC) generation and self-renewal, T and B lymphocyte development, and differentiation from HSCs, which provides novel insights into the role of m^6^A as a critical regulator of the immune system.

## 2 The process, hub regulators, and the mechanism of m^6^A modification

**Figure 1:**
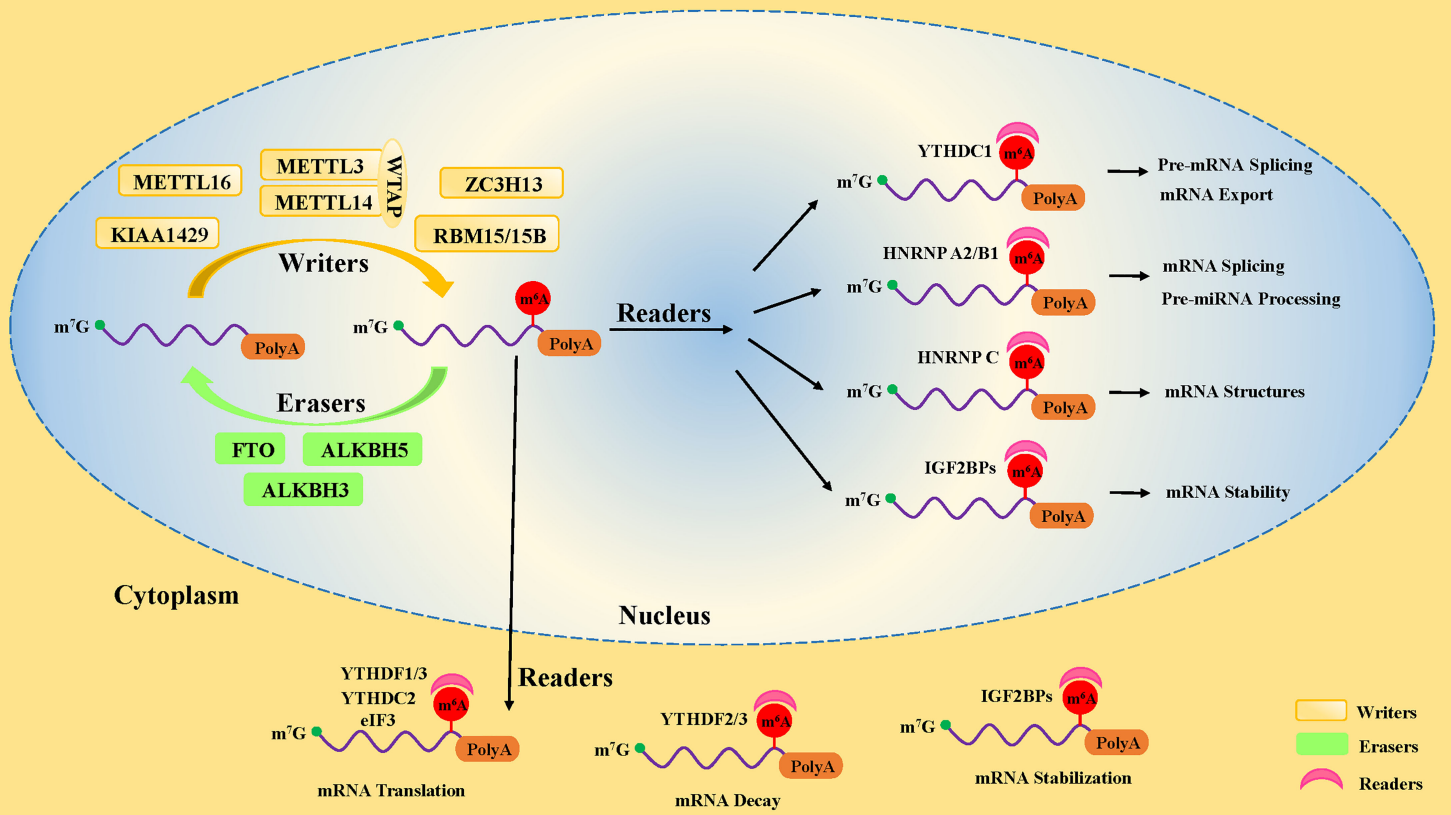
The dynamic process of m6A methylation in mRNA. The m^6^A methylation is installed by methyltransferases (“writers”), including METTL3, METTL14, WTAP, METTL16, ZC3H13, RBM15/15B, and KIAA1429. It is removed by demethylases (“Erasers”), including FTO, ALKBH5, and ALKBH3. Moreover, the fate of m^6^A-modification RNA is determined by RNA-binding proteins (“readers”), including YTHDF1, YTHDF2, YTHDF3, YTHDC1, YTHDC2, IGF2BPs, HNRNP A2/B1, and eIF3.

Studies have uncovered that the motifs of m^6^A modification in RNA sequences are widespread and highly conserved in eukaryotes, mainly occurring on the following consensus sequence: RRACH (R∶G or A; H=A, C, or U) (12, 13). In pre-mRNA, m^6^A modification motifs are highly enriched near the splicing sites and the last exon, while in mature mRNA, they are mainly distributed in the translation start sites, coding sequences (CDS), and 3’-untranslated region (3’UTR), especially near the stop codon and within internal long exons (5, 6). There are three kinds of pivotal protein factors involved in m^6^A methylation, including methyl-transferases (writers), demethylases (erasers), and methylation binding protein (readers) (14) (**Figure 1**).

### 2.1 Methyl-transferases, the m^6^A writers, mark the RNA with the methyl group

The first protein that catalyzes the methylation of adenosine on RNA is a methyl-transferase complex (15). It has been established that methyl-transferase-like 3 (METTL3), methyl-transferase-like 14 (METTL14), and Wilms tumor 1-associated protein (WTAP) are the core components of the methyl-transferase complex that promote the incorporation of the m^6^A methylated group into RNA (16–18) (**Figure 1**). In addition to the core components, many other proteins, including METTL5 (19) and METTL16 (20), Zinc Finger CCHC-Type Containing 4 (ZCCHC4) (21), Zinc Finger CCCH-Type Containing 13 (ZC3H13) (22, 23), KIAA1429 (24), and RBM15 (and its homolog RBM15B) (9), have been found to participate in the formation of the methyl-transferase complex, playing different roles in methylation modifications of human pre-mRNAs and various non-coding RNAs (25, 26).

METTL3 and METTL14 form a 1:1 stable heterodimer complex in the nucleus (27). METTL3 is a molecule with catalytic activity, while METTL14 is a binding protein that acts as a scaffold for mRNA binding (28). METTL3 could modify adenosine196 (A_196_) in the 5’-external transcribed spacer of 47S pre-rRNA, leading to decreased rates of pre-rRNA maturation (29). In the nucleus, the METTL3/14 heterodimer interacts with WTAP. It has been reported that WTAP has no methyl-transferase activity, regulates the binding of METTL3/14 complex to transcription sites, and recruits the heterodimers to nuclear speckles (18, 27). Knockdown of WTAP results in attenuation of the interaction between the METTL3/METTL14 complex and mRNA and reduced localization of the complex on the subcellular organelle nuclear plaques of mRNA alternative splicing and ribonucleoprotein assembly (18). WTAP also recruits other related factors, such as RBM15 and KIAA0853, to the methyl-transferase complex, regulating the methylation activity (30). These pieces of evidence indicate that WTAP is critical for m^6^A methylation activation.

It has been shown that METTL5 forms a heterodimeric complex with the methyl-transferase activator TRMT112 through the formation of a parallel β-zipper between the main chain atoms and is responsible for installing m^6^A at A_1832_ on 18S rRNA, thus regulating the translation process (19). METTL16 can reportedly act as a triple-stranded RNA binding protein and support the formation of lncRNA triple helices (20). In addition, under S-adenosylmethionine (SAM)-limiting conditions, METTL16 occupancy on the hairpin (hp1) of the MAT2A 3’-UTR is increased, thus promoting MAT2A splicing (25). ZCCHC4 accumulates in the nucleolus, and the methyl-transferase (MTase) domain of ZCCHC4 is packed against N-terminal GRF-type and C2H2 zinc finger domains and a C-terminal CCHC domain, creating an integrated RNA-binding surface (31). Then, ZCCHC4 deposited m^6^A at A_4220_ of human 28S rRNA, which impacts ribosome subunit distribution and global translation (19, 21). In the nucleus, ZCCHC13 was reportedly essential for nuclear localization of WATP, KIAA1429, and HAKAI to facilitate m^6^A methylation of mRNA (23).

### 2.2 M^6^A erasers remove the methyl groups from RNA by oxidative demethylation

The modification of m^6^A is a dynamic reversible process, and the m^6^A methylated groups on RNA can be removed by demethylase. Till now, three kinds of m^6^A demethylases, Fat mass and obesity-associated protein (FTO), ALKB homolog 5 (ALKBH5), and ALKB homolog 3 (ALKBH3), have been identified (32, 33) (**Figure 1**). All of them belong to the ferrous iron and α-ketoglutarate (αKG)-dependent dioxygenase ALKB family. However, the expression and cellular localization of FTO and ALKBH5 are different in diverse tissues, indicating that they possess many biological functions. FTO was the first identified m^6^A demethylase in 2011 (32). FTO knockdown with siRNA in HeLa and 293FT cells resulted in increased methylation levels *in vitro*, while the m^6^A level of mRNA was decreased by FTO overexpression using a mammalian expression vector in HeLa cells (32).

Varying subcellular localization of FTO is associated with different catalytic substrates. FTO located in the nucleus has been reported to catalyze the mRNA demethylation of m^6^A, while FTO located in the cytoplasm preferentially demethylates the m_2_ isoform m^6^Am (N6-2’-O-dimethyladenosine) of mRNA (34). In addition, cytoplasm FTO demethylates m^6^A in U6 RNA and m^6^Am in snRNAs. FTO mediates m^1^A demethylation in tRNA (34). Interestingly, FTO can reportedly oxidize m^6^A to form two intermediates, N^6^-hydroxymethyladenosine (hm^6^A) and N6-formyladenosine (fm^6^A), with a half-life of 3 h in the nucleus, which may dynamically modulate RNA–protein interactions (35).

Another demethylase ALKBH5 specifically and directly catalyzes the demethylation of m^6^A without any intermediate (35, 36). According to a recent study in 2013, ALKB homolog 3 (ALKBH3) is also an m^6^A demethylase (33). However, ALKBH3 preferentially modifies tRNA, not mRNA or rRNA, to enhance protein translation efficiency by extending tRNA’s half-life (37). Whether other ALKB protein family members possess demethylase activity remains poorly understood, warranting further clarification.

### 2.3 M^6^A readers determine the fate of m^6^A-modified RNA

The third most important factor of m^6^A modification is the RNA-binding proteins called “readers”. They properly decode the m^6^A RNA methylation information in cells by recognizing and binding m^6^A-modified RNA. Moreover, they are involved in the regulation of RNA processing and metabolism (**Figure 1**), such as RNA degradation (38), alternative splicing (39), and translation (40). The earliest identified readers were the YT521-B homology (YTH) domain family proteins in the mammals, consisting of the YTH domain family (YTHDF) and the YTH domain-containing protein (YTHDC) subsets, including YTHDF1, YTHDF2, YTHDF3 (38), YTHDC1, and YTHDC2 (41).

YTHDF subtypes are mainly found in the cytoplasm (42). Different YTHDF readers have different biological effects through dissimilar pathways. YTHDF1 improves the efficiency of m^6^A-containing mRNA translation by interacting with eukaryotic initiation factor 3 (eIF3) and contributing to ribosome occupancy of target mRNA (40), while YTHDF2 catalyzes mRNA degradation by shortening the half-life of mRNA in the cytoplasm (43). Two different research teams have confirmed that YTHDF3 interacting with YTHDF1 and YTHDF2 enhanced the YTHDF1-facilitated translation of methylated mRNA and promoted YTHDF2-mediated mRNA decay (44, 45). This finding suggests that these three YTHDF proteins work synergistically in the regulation m^6^A-modified mRNA modulation (44, 45). Moreover, it may also imply that YTHDF2 promotes genes expression in the short term while shortening the subsequent effects, which may benefit the cell response to the environment under stress conditions. However, Du et al. discovered that these three types of YTHDF proteins function similarly on mRNA degradation (43), probably because of their high homology. Intriguingly, the YTHDF2 protein has been reported to promote cap-independent mRNA translation in MEF cells in response to heat shock stress, since YTHDF2 transferred from cytoplasm to nucleus was found to compete with FTO in preserving 5’-UTR methylation of stress-induced mRNA (46). The 5’-UTR translation initiation codon is known to mediate the initiation of translation of eukaryotic mRNA (47). Moreover, it has been shown that YTHDF2 binds directly to 5-methylcytosine in rRNA and modulated the maturation of rRNA (48).

YTDHC1 has been established to be only located in the nucleus, regulating mRNA expression levels (39) and mRNA exporting from the nucleus by influencing alternative splicing (49). In addition, YTDHC1 is required for sufficient rRNA synthesis *via* regulating the scaffold function of long interspersed nuclear element-1 (LINE1) RNA (50). YTDHC2 exists in the nucleus and cytoplasm, which improves the translation efficiency of target mRNA in mice spermatogenesis (51), supporting its testicular special function. However, gene database analysis revealed that YTDHC2 is moderately expressed in immune cells, which indicates potential existence of an undocumented role in immune cells (52).

Several researchers have reported that some additional RNA-binding proteins, such as eIF3, HNRNP family proteins (including HNRNP A2/B1, HNRNP C, HNRNP G), and insulin-like growth factor 2 mRNA-binding proteins (IGF2BPs, including IGF2BP1, IGF2BP2, and IGF2BP3), also function as “readers” to decode the m^6^A RNA methylation information. EIF3 binds to mRNA in the 5’-UTR region and mediates the initiation of mRNA translation (53). HNRNP A2/B1 was reportedly involved in alternative splicing of mRNA and processing of precursor miRNAs in the nucleus (54, 55). However, HNRNP C preferentially bound to m^6^A-modified sites, weakening the capability of mRNAs and long non-coding RNAs (lncRNAs) to form local secondary structures in the nucleus (56). Little is known about HNRNP G and its role in m^6^A modification. IGF2BPs have been reported to promote the stability and storage of their target mRNAs in an m^6^A-dependent manner, thus enhancing target mRNA expression in the nucleus and cytoplasm (57). In recent years, new writer and reader proteins involved in m^6^A modification are continued to be discovered, suggesting that the biological effects and significance in potentially regulating the m^6^A modification remain poorly understood, leaving a broad area of research to explore.

## 3 Techniques for m^6^A detection in RNA

M^6^A modifications have been found in mRNA as early as the 1970s (4, 58). However, due to technical limitations, researchers could not detect m^6^A, especially quantify m^6^A levels, and let alone identify m^6^A from the single base level, which retarded the progress of scientific studies in this field for a long time. With the rapid development of next-generation sequencing (NGS) technologies and the improvement of liquid chromatography sensitivity (6), scientists have developed various m^6^A detection methods.

At present, to detect m^6^A, high-throughput sequencing is used, such as methylated RNA immunoprecipitation with next-generation sequencing (MeRIP-seq), m^6^A individual-nucleotide-resolution cross-linking and immunoprecipitation with sequencing (miCLIP-seq), ligation-assisted extraction and thin-layer chromatography (SCARLET), and liquid chromatography-mass spectrometry (LC-MS/MS). MeRIP-seq enables qualitative analysis of the hypermethylated mRNA region, while single-base resolution is not feasible (5). However, miCLIP-seq and SCARLET have been reported to accurately locate m^6^A loci in the whole transcriptome at a single-nucleotide resolution level (59, 60), while LC-MS/MS could detect the overall m^6^A level of mRNA (61).

## 4 The Role of m^6^A modification in the hematopoietic stem cells, and T and B lymphocytes

**Figure 2:**
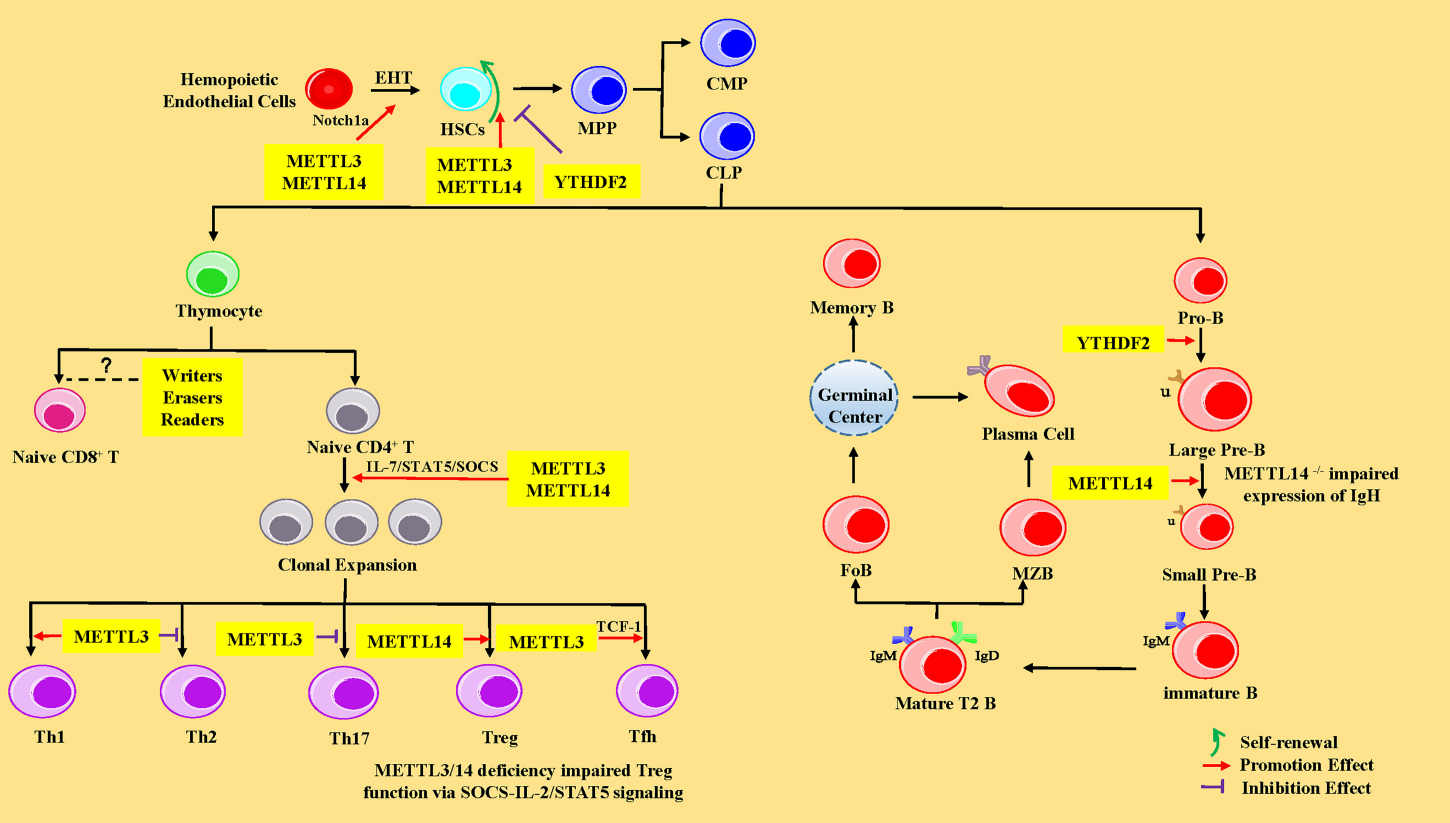
The regulatory role of m6A modification in HSCs, and T and B lymphocytes. In the HSCs, METTL3 and YTHDF2 promote endothelial-to-hematopoietic transition (EHT) *via* Notch1a leading to HSC generation. METTL3 and METTL14 enhance the ability of HSC self-renewal through MYC, whereas YTHDF2 inhibits HSC self-renewal by promoting the decay of mRNA encoding transcription factors, including TAL1, GATA2, RUNX1, and STAT5. MPP, multipotent progenitor cells; CMP, common myeloid progenitors; CLP, common lymphoid progenitors. In CD4^+^ T cells, METTL3 and METTL14 promote naive CD4^+^ T-cell proliferation by the IL-7/STAT5/SOCS pathway. Meanwhile, METTL3 promotes the differentiation of Th1 and Tfh cells, but represses Th2 and Th17 cell differentiation. METTL14 promotes Tregs differentiation, and METTL3/14 is essential for the suppressive function of Tregs. However, the exact roles of m^6^A modification in CD8^+^ T cells is unknown. In B cells, YTHDF2 and RBM15 promote pro-B-to-large-pre-B transition, and METTL14 promotes the transition from large pre-B cells to small pre-B cells *via* increasing chromatin accessibility of key transcription factors loci (*Ikzf3, Irf4, Spib*, and *Bcl6*). However, loss of METTL14 did not affect IgH recombination, but might impair the expression of recombined IgH. IGF2BPs promote the differentiation of MZB and FoB by enhancing the stability of B-cell regulators Pax5 and Arid3a mRNA. Pro-B, progenitor B cells; Large Pre-B, large precursor B cells; Small Pre-B, small precursor B cells; MZB, marginal zone B cells; FoB, follicular B cells.

HSCs are multipotent cells with the lifelong ability to self-renew and can differentiate into all of the cells of the blood and immune system. In vertebrates, HSCs are derived from hematopoietic endothelial cells (HECs) through endothelial-to-hematopoietic transition (EHT) in the aorta-gonad-mesonephros (AGM) region during the embryonic development stage. Subsequently, HSCs migrate to the fetal liver for massive amplification and transfer to bone marrow (BM) after birth (62). In the BM, HSCs give rise to multipotent progenitor cells (MPPs), consisting of common myeloid progenitors (CMPs, with myeloid, erythroid and megakaryocytic potential) and common lymphoid progenitors (CLPs, with only lymphoid potential) (63). CMPs eventually differentiate into neutrophils, macrophages, eosinophils, basophils, erythroid cells, and monocytes, whereas CLPs subsequently develop and differentiate into T lymphocytes, B lymphocytes, and natural killer cells (NK cells) (63). Recent studies have shown that m^6^A-mediated various RNA metabolic processes are involved in the stepwise differentiation process (**Figure 2**). The following sub-chapters introduce more specific details of the role of the RNA m^6^A modification and its associated regulatory proteins in the HSC generation and self-renewal, cell development, and differentiation of T and B lymphocytes from HSCs.

### 4.1 The m^6^A modification in hematopoietic stem cells

#### 4.1.1 M^6^A modification promotes HSC generation *via* degrading Notch1a

In 2017, researchers found that METTL3 was abundantly expressed in endothelial cells and HECs in zebrafish, indicating that the function of METTL3 was closely related to EHT (7). Previous studies have elucidated that the inhibition of Notch signaling in endothelial cells promotes EHT and the generation of HSCs (64, 65). Once METTL3 was deleted in the embryos of zebrafish with the CRISPR-Cas9 system or in the endothelial cells of mice AGM region using the Cre/Loxp system, the mRNA level of Notch1a was significantly increased, leading to activation of Notch signaling, thus significantly repressing the EHT process and hindering the generating of HSCs (7, 66) (**Figure 2**). The aforementioned phenomenon could be reversed by forcing METTL3 expression in endothelial cells (7). METTL14 is a heterologous partner of METTL3 (27), implying the importance and role of METTL14 in hematopoiesis. Furthermore, the YTHDF2 recognizes the m^6^A peak near the stop codon of Notch1 mRNA (7, 66) and mediates Notch1a mRNA decay (67). Interestingly, the YTHDF2 morphants in zebrafish and mice conferred a similar phenotype in METTL3-deficient embryos *via* mediating Notch1a mRNA decay (7). In summary, the regulation of m^6^A on HSC specification is evolutionally conserved during EHT in vertebrates and mainly influences the stability of Notch1a.

#### 4.1.2 M^6^A modification regulates self-renewal and lineage differentiation of HSCs by affecting the half-life or translation of MYC mRNA

HSCs are well-characterized with the ability for self-renewal and multi-lineage differentiation. Recent evidence has revealed that m^6^A modification at the mRNA level is a new way to regulate HSC self-renewal and differentiation. The transcripts of m^6^A “writers” METTL3 and METTL14 were abundant in mouse HSCs and significantly downregulated in more mature committed CMPs, especially in myeloid cells (68–70). METTL3 governed the abundance of MYC in HSCs at a decision point that dictates the choice between self-renewal versus differentiation (71). The deletion of METTL3 with Mx1-cre downregulated MYC expression *via* regulating mRNA translation rather than changes in MYC transcript levels, contributing to an accumulation of HSCs in the BM and a marked reduction of reconstitution potential due to a symmetrical differentiation defects *in vivo* (70, 71). However, short hairpin RNA (shRNA)-mediated depletion of METTL3 in human umbilical cord blood (hUCB)-derived HSCs and human myeloid leukemia cell line (68) or knocking down of METTL14 in primary leukemic blasts (CD34^+^) resulted in myeloid differentiation with increasing phosphorylated AKT levels and the inhibition of cell growth with the reduction of colony formation *in vitro* (68, 69). It is well-recognized that the development and differentiation of HSCs *in vivo* are not only regulated by cell-intrinsic genes, but also affected by external factors, such as hematopoietic microenvironment. A study revealed that the self-renewal ability of HSCs predominantly depends on METTL3, not on METTL14 (72). In addition, METTL3 depletion in HSCs did not affect apoptosis (68), whereas knocking down of METTL14 induced acute myeloid leukemia (AML) cell apoptosis and promoted myeloid differentiation of normal HSCs *via* the SPI1–METTL14–MYB/MYC signaling axis (69).

RBM15 is another important component of m^6^A “writers”, originally found to be involved in hematopoiesis. RBM15 has been shown to exhibit high expression in HSCs. The deletion of RBM15 increased the number of HSCs in RBM15^flox/null^ Mx1-Cre BM, and RBM15-deficent HSCs showed a shift in progenitor fate toward granulocyte differentiation or favored megakaryocyte development with abnormally small and low-ploidy megakaryocytes *via* downregulation of c-MYC (73, 74). Furthermore, RBM15 knockdown with RNA interference in 32DWT18 myeloid precursor cell line enhanced myeloid differentiation *via* a mechanism mediated by Notch signaling stimulation *via* altering recombination signal-binding protein for immunoglobulin kappa J region (RBP Jκ) and hairy and enhancer of split homolog-1 (HES1) promoter activity (75).

Furthermore, one research team used Mx1-Cre mice to achieve m^6^A “reader” YTHDF2 conditional knockout mice and observed that deletion of YTHDF2 in HSCs led to a significant increase in the absolute numbers as well as the frequency of functional HSCs with normal lineage differentiation *in vivo via* the activation of WNT signaling downstream targets including MYC, CCND1, and AXIN2 (76). In addition, another study revealed that lentivirus mediated-knockdown of YTHDF2 in human CD34^+^ HSCs resulted in expansion *ex vivo.* However, in molecular mechanism, YTHDF2 deficiency promotes the decay of mRNAs that encode transcription factors involved in stem cell self-renewal, including TAL1, GATA2, RUNX1, and STAT5 (76, 77). However, there was no significant difference in the absolute number of progenitors (such as MPPs, CMPs, and CLPs), as well as myeloid cells, B cells, and T cells (76, 77). In a nutshell, the above studies corroborated that MYC is a critical target of m^6^A modification in HSCs. M^6^A modification-related proteins can regulate the self-renewal and differentiation of HSCs by affecting the half-life or translation of MYC mRNA.

HSC transplantation is widely used to treat a broad spectrum of disorders, such as hematologic diseases, immune disorders, and cancer. However, the inability to expand HSCs *in vitro* hampers their potential clinical application. Accordingly, the expansion of HSCs remains a conundrum, warranting further investigation. Recent studies have clarified that m^6^A modification regulates the self-renewal of HSCs. Thus, the self-renewal and induced expansion of HSCs *in vitro* after treatment with specific inhibitors or activators of m^6^A are widely believed to provide an adequate source for the clinical application of HSCs in the future.

### 4.2 The m^6^A modification in T cells

HSCs can differentiate into CLPs in the BM, then migrate into the thymus through blood circulation in which they undergo gene rearrangement to express diverse TCR and become mature CD4^+^ or CD8^+^ naive T cells through negative and positive selections (78). After that, naive T cells are driven into peripheral immune organs where naive CD4^+^ T cells, activated by antigens, eventually differentiate into distinct effector T cells sub-populations (Th1, Th2, Th17, Treg, and Tfh) or memory T cells. At the same time, the naive CD8^+^ T cells develop to cytotoxic T cells (CTL) after recognizing the antigens by antigen-presenting cells (79).

#### 4.2.1 M^6^A modification plays a dispensable role in the development of T cells and promotes T-cell proliferation *via* the IL-7/STAT5/SOCS pathway

Most recent studies have revealed that m^6^A methylation is critical in the development, proliferation, and functional performance of CD4^+^ T cells. The numbers of naive T cells were increased in the spleen and lymph nodes but not in the thymus in CD4-Cre conditional mice with CD4^+^ T cell-specific deletion of the writer protein METTL3 or METTL14 (80). However, METTL3^-/-^ or METTL14^-/-^ naive T cells remained in the naive “progenitor” state for more than 4 weeks (80). Similarly, the development of T cells was not affected in the thymus and peripheral lymphoid tissues of CD4^+^ T cell-specific eraser protein ALKBH5-deficient mice in the steady state (81), suggesting a dispensable role for m^6^A in the development of T cells (**Figure 2**).

The mRNA level of ALKBH5 was upregulated after activation of T-cell receptor (TCR) signaling, while the expression of FTO mRNA exhibited no evident change (81). Interestingly, it was puzzling that lack of ALKBH5 did not affect the activation, proliferation, apoptosis, and cytokine secretion of T cells *in vivo* (81). It is well established that elevated levels of IL-7 can induce proliferation of naive T cells in lymphocytopenia mice after adoptive transfer (82), and SOCS proteins can repress IL-7/JAK/STAT signaling and play an important role in T-cell proliferation and differentiation (83–87). The mRNA 3’-UTR and 5’-UTR of suppressor of cytokine signaling (SOCS) family genes (*SOCS1*, *SOCS3*, and *CISH*) have specific m^6^A peaks, with a conservative sequence of GG/AACA/U (80, 88). Loss of METTL3 increased the expression of SOCS, especially SOCS1, *via* increasing the mRNA half-life and decreasing m^6^A-mediated degradation, leading to the inactivation of the IL-7/STAT5/SOCS pathway (80). Hence, researchers found that T cells lacking METTL3 or METTL14 failed to proliferate using the adoptive transfer model *in vivo* (80, 89). In summary, m^6^A modification promotes T-cell proliferation *via* the IL-7/STAT5/SOCS pathway.

#### 4.2.2 The effect of m^6^A modification on the differentiation of Th1, Th2, and Th17 cells

Naive T cells can differentiate into distinct T helper effector cell subsets and perform different functions after stimulation by cytokines *in vitro* and various antigens *in vivo*. Recent studies have illustrated that m^6^A methylation plays a crucial regulatory role in T-cell differentiation. The deletion of METTL3 in the CD4^+^ T cells reinforced Th2 cell differentiation (80) but repressed Th1 and Th17 cell differentiation (80, 90). However, METTL3 deficiency did not affect Th2 cell differentiation following keyhole limpet hemocyanin (KLH) immunization in METTL3-deficient mice (90). Moreover, mice with specific deletion of METTL14 in CD4^+^ T cells developed spontaneous colitis, characterized by increased inflammatory cell infiltration along with a marked increase of Th1 cytokines (IFN-γ and TNF-α) and Th17 cytokines (IL-17a and IL-17c) in colonic epithelial (91). In contrast, the level of IL-25 produced by Th2 cells was dramatically reduced, while IL-13 levels did not change (91). Moreover, researchers uncovered that the expression of ALKBH5 mRNA was increased, and there were no evident changes in the expression of FTO mRNA in Th1, Th2, Th17, and regulatory T cells (Tregs) compared to naive CD4^+^ T cells (81). Mice with CD4^+^ T cell-specific deletion of ALKBH5 were resistant to autoimmune colitis and experimental autoimmune encephalomyelitis (EAE) induction with diminished recruitment of neutrophils into the central nervous system. This phenomenon was explained by the fact that ALKBH5 ablation decreased the mRNA stability of CXCL2 and IFN-γ by enhancing RNA decay and promoted CD4^+^ T-cell pathogenicity (81). Unlike ALKBH5, ablation of FTO did not impair T-cell development or promote EAE pathogenesis (81). One possibility can be advanced that lack of FTO may result in compensation response by other epigenetic regulation.

#### 4.2.3 M^6^A modification regulates the immunosuppressive function of Tregs *via* SOCS-IL-2/STAT5 signaling

Tregs, the critical specialized T cell subset, are involved in reducing inflammation and immunosuppression by producing anti-inflammatory cytokines such as IL-10 and TGF-β, regulating the activity of various immune cells, and eventually suppressing the immune response and guarding against autoimmune diseases (92). The results from three research groups substantiated that METTL3 had no significant regulatory effect on directing Treg cell differentiation (80, 88, 89) (**Figure 2**). Similar to METTL3, loss of YTHDF1 yielded no significant difference in the number of Tregs (93). However, mice lacking METTL3 in CD4^+^ T cells and Tregs developed chronic intestinal inflammation and alopecia, respectively. These severe autoimmune diseases were attributed to an absolute lack of immunosuppressive function for Tregs (80, 88). The IL-2/STAT5 pathway was crucial for Tregs function and stability (94). The researchers used m^6^A irCLIP-Sep technology (a UV-C crosslinking and immunoprecipitation platform) and revealed that m^6^A modifications were indeed enriched at 3’-UTR and 5’-UTR regions of SOCS genes, with a conservative sequence of GG/AACA/U (88). It has been elicited that deletion of METTL3 increased the levels of SOCS proteins by enhancing SOCS mRNA stability (80, 88), thereby suppressing the IL-2/STAT5 signaling pathway (80, 88). Additionally, deficiency of another methyl-transferase METTL14 in mice blocked the differentiation of naive T cells into induced Tregs and resulted in Tregs dysfunction with loss of suppressive capacity, which was supported by the fact that the METTL14-deficient Tregs were unable to suppress naive T cell-induced colonic inflammation (91). However, m^6^A demethylase ALKBH5 deficiency in Tregs exerted no effect on immunosuppressive function (81). The role of RNA binding proteins in Tregs has not been reported, which could be future directions for investigation.

#### 4.2.4 M^6^A modification promotes Tfh cell differentiation *via* the METTL3–TCF-1 axis

T follicular helper (Tfh) cells, a specialized subset of CD4^+^ T cell located within germinal centers (GC) of lymph nodes, are involved in the development of humoral immunity by controlling the GC formation and B-cell responses, especially in the differentiation of B cells into plasma cells, antibody production, and Ig class switch (95). T cell-special transcription factor 1 (TCF-1), a crucial transcription factor for Tfh cells differentiation, is encoded by the *Tcf7* gene (96, 97). Tcf7 is a bona fide m^6^A target. METTL3 directly binds to the 3’-UTR region of TCF-1 mRNA to slow down the degradation and enhance its stability, ensuring TCF-1 protein expression (90). Therefore, the knocking down of METTL3 or METTL14, rather than ALKBH5, with shRNA in CD4^+^ T cells could promote Tfh cell development after lymphocytic choriomeningitis virus (LCMV) infection (89). However, conflicting findings were reported by Yao et al. that the conditional deletion of METTL3 with CD4-cre in CD4^+^ T cells severely impaired Tfh cell differentiation in a cell-intrinsic manner after LCMV infection (90). This inconsistency may be accounted for by the heterogeneity in experimental systems. Meanwhile, METTL3 deficiency in CD4^+^ T cells reduced the frequency and cell numbers of GC B cells and plasma cells and METTL3^-/-^ mice exhibited a significantly lower concentration of the LCMV-specific IgG, indicating that the function of Tfh cells was impaired (90).

To sum up, the METTL3–TCF-1 axis functions as an important regulator to initiate and ensure the differentiation of Tfh cells post-transcriptionally. Further efforts are needed to investigate whether other m^6^A modified proteins are involved in Tfh cell differentiation.

### 4.3 The m^6^A modification in B cells

B-cell development represents a highly ordered process that involves sequential immunoglobulin gene recombination. In the BM, CLPs give rise to immature B cells that undergo stepwise differentiation stages from progenitor B cells (pro-B cells) to precursor B cells (pre-B cells), and to immature B cells. Subsequently, the immature B cells further mature in the spleen (98, 99). Pre-B cells can be divided into two subsets: the immature, actively dividing large pre-B cells and the more mature, quiescent small pre-B cells (100). These stages are defined by rearranging the gene’s loci encoding immunoglobulin (H and L chain) and the expression of differentiation-specific molecules on the cell surface. Pro-B cells undergo VDJ gene recombination mediated by recombination activating genes (RAG) and express surface molecules such as CD19. Once the μ heavy chain (Igμ) is expressed, the cell becomes a pre-B cell. Pre-B cells express a set of B lineage-specific genes called λ5 (CD179b) and VpreB (CD179a), which form an IgL chain-like structure known as the surrogate light chain (SLC) to pair with the Igμ heavy chain to combine to form a functional pre-B cell receptor (pre-BCR) (101, 102). Furthermore, IL7 is a pre-B cell stimulant (103), and pre-BCR enhances the reactivity of pre-B cells to IL-7 and promotes clonal expansion along with IL-7R (100). Then, the complete IgM molecule (BCR) formed by V to J rearrangements at the κ and λ light chain gene loci is expressed on the cell surface, indicating that the cells have developed into immature B cells. Lastly, immature B cells migrate into the spleen to mature and become transitional type 1 and type 2 B lymphocyte subsets (T1 and T2) (104). T2 B cells have been established to differentiate into follicular B cells (FoB) or marginal zone B cells (MZB) (104). Upon encountering antigen, mature B cells are activated and enter the GC, undergoing rapid growth and proliferation. Furthermore, GC B cells differentiate into either antibody-secreting plasma cells or long-lived memory B cells (105).

#### 4.3.1 M^6^A modification controls early B cell development

More recently, it has been demonstrated that RNA m^6^A modification plays a pivotal regulatory role during early B-cell development. Two recent studies reported that there were no major defects in the BM B cells after poly I:C treatment-induced METTL3 deletion, which might be explained by two hypotheses: deletion of METTL3 was incomplete, or METTL3 was dispensable for the maintenance and survival of B cells (70, 71). However, METTL14 deficiency has been shown to block the transition from large pre-B cells to small pre-B cells *in vivo* and *in vitro* (106). Moreover, METTL14 deficiency did not affect IgH recombination but might impair the expression of recombined IgH (106). METTL14-deficient B cells showed limited ability to rearrange Igκ or downregulate the pre-BCR component VpreB (106). Meanwhile, deletion of YTHDF2 resulted in a significant block between the pro-B stage and the late large pre-B stage *in vivo* (106) (**Figure 2**). However, YTHDF1 is not essential for B-cell development (106). These developmental defects were attributed to the decreased chromatin accessibility of key transcription factors loci (*Ikzf3*, *Irf4*, *Spib*, and *BCL6*) mediating the large-pre-B-to-small-pre-B transition and BCR recombination components (Rag1 and Rag2), thus resulting in the fact that these key transcription factors could not be transcribed (106). RBM15^flox/null^ Mx1-Cre mice treated with polyinosinic-polycytidylic acid (PIC) showed a dramatic decrease in peripheral B cells due to a block in pro/pre-B differentiation (73). However, the percentages of IgM^+^ IgD^+^ and IgM^-^ IgD^+^ B cells were maintained in peripheral blood, which suggested that RBM15 is not essential for B cells in the GC (73) (**Figure 2**). Additionally, IGF2BP3 forced expression with transduction of FH-IGF2BP3-RV in HSCs increased the frequency of MZB and FoB by enhancing the stability of B-cell regulators Pax5 and Arid3a mRNA (107).

CD40 (also called TNFRSF5) is a member of the tumor necrosis factor receptor (TNFR) superfamily with essential roles in B-cell development, activation, GC formation, and class-switched antibodies (108, 109). Notably, the m^6^A writer WTAP and the m^6^A reader YTHDF2 are key suppressors of CD40 (110). However, little is known about the role of WTAP in B cell development.

#### 4.3.2 The effect of m^6^A on B cell activation and proliferation

Furthermore, IL-7 induces the proliferation and differentiation of pro-B cells to pre-B cells (103). After IL-7 stimulation, loss of METTL14 impaired pro-B-cell proliferation and cell size enlargement *in vitro* and led to significantly lower proliferation rates in pro-B cells and the early large pre-B cells *in vivo*, consistent with the observation in the YTHDF2-deficient B cells *in vitro* (106). These findings strongly suggest that the mRNA m^6^A methylation is important for the IL-7-induced pro-B-cell proliferation by promoting the decay of a group of YTHDF2-bound transcripts (106). Diffuse large B-cell lymphoma (DLBCL) is the most common lymphoid malignancy derived from germinal center B cells with malignant proliferation (111). Cheng et al. revealed that METTL3 expression was increased both in lymph nodes from DLBCL patients and in DLBCL cell lines, including SU-DHL4, OCILy10, Farage, U2932, and HBL1. Silencing METTL3 using lentivirus-mediated shRNA in cell lines inhibited cell proliferation by abating the total mRNA level of pigment epithelium-derived factors (PEDF), which was usually regarded as a canonical WNT signaling inhibitor. However, WNT/β-catenin signaling was not activated as a result (112). These findings suggest that MEETL3 may be involved in B-cell proliferation.

Taken together, we conclude that m^6^A modification controls early B-cell development and IL-7-induced pro-B-cell proliferation. Impairment of m^6^A modification hinders B-cell proliferation, development, and maturation.

## 5 Conclusion

The physiological and pathological function of m^6^A modification has become an emerging field of investigation since discovering m^6^A modification on RNA. Over the past decade, ample evidence has corroborated that m^6^A modification is involved in the development, differentiation, and function of many immune cells. In this review, we summarize the components of m^6^A regulators (**Figure 1**) and recent findings of m^6^A modification from HSCs to T and B lymphocytes.

We provided a comprehensive overview that m^6^A modification regulates the generation, self-renewal, and lineage differentiation of HSCs; governs CD4^+^ T-cell development, activation and clonal proliferation, differentiation, and subsequent effector functions; and controls B cells’ early development, activation, and proliferation through different mechanisms. Moreover, we summarize the molecular mechanisms involved in m6A modification in HSCs, T and B lymphocyte development. These molecules are recognized by the RNA methylation readers and are degraded or translated subsequently. Nevertheless, it remains unknown how m^6^A modification functions precisely in some special T-cell and B-cell subset, such as Th9 cells, memory CD4^+^ T cells, Breg cells, plasma cells, and memory B cells, nor is it clear whether m^6^A modifications regulate the development and function of CD8^+^ T cells, warranting additional investigation (**Figure 2**).

Current studies have merely revealed m^6^A modifications on mRNA in immune cells and overlooked the effect of m^6^A modification on tRNA and rRNA. M^6^A modification may regulate the synthesis of proteins that play an important role in the development of immune cells by influencing ribosome occupancy and translation efficiency. Overall, m^6^A methylation in immune cells is a new research hotspot with more exciting discoveries expected in the future. Indeed, m^6^A methylation modification could be a novel therapeutic target in alleviating immune cell-related inflammatory diseases and infections and promoting cancer immunotherapy. For example, m^6^A small-molecule drugs are used to treat HSCs to enhance the self-renewal ability of HSCs and can yield a large number of HSCs *in vitro*, which provide sufficient HSCs for stem cell transplantation. Moreover, it should be borne in mind that selective deletion of m6A in tumor-infiltrated Tregs may abate the inhibitory function, thereby recovering the tumor-killing functions of CD8^+^ T cells. Furthermore, the deficiency of m^6^A in Tfh cells and the enhancement of m^6^A in Tregs reduce the number of plasma cells and the production of autoantibodies and increase immunosuppressive function for Tregs, representing a promising therapeutic strategy against autoimmune diseases.

## Author contributions

CZ wrote the original draft. QS, GX, and XZ provided suggestions about the structure of the manuscript and the writing of molecular mechanisms of M^6^A modification in the immune system and summarized relevant studies. WC and QS helped to organize and revise the manuscript. QS, CZ, GX, and YY were the funding recipients. All authors contributed to the article and approved the submitted version.

## Funding

Our work was supported by grants from the National Natural Science Foundation of China (Grant No. 82071738), Huai’an “Tianyixing” key Laboratory of Medical Examination (Grant No. HAP202004), Huai’an Natural Science Research Program (Grant No. HABL202114), and the Medical Leadership Program of Jiangsu College of Nursing (Grant No. 2021001).

## Conflict of interest

The authors declare that the research was conducted in the absence of any commercial or financial relationships that could be construed as a potential conflict of interest.

## Publisher’s note

All claims expressed in this article are solely those of the authors and do not necessarily represent those of their affiliated organizations, or those of the publisher, the editors and the reviewers. Any product that may be evaluated in this article, or claim that may be made by its manufacturer, is not guaranteed or endorsed by the publisher.
